# Transcriptome analysis of two buffalograss cultivars

**DOI:** 10.1186/1471-2164-14-613

**Published:** 2013-09-11

**Authors:** Michael Wachholtz, Tiffany Heng-Moss, Paul Twigg, Lisa Baird, Guoqing Lu, Keenan Amundsen

**Affiliations:** 1 Department of Biology and School of Interdisciplinary Informatics, University of Nebraska at Omaha, Omaha, NE 68182, USA; 2 Department of Entomology, University of Nebraska at Lincoln, Lincoln, NE 68583, USA; 3 Department of Agronomy & Horticulture, University of Nebraska at Lincoln, Lincoln, NE 68583, USA; 4 Department of Biology, University of Nebraska at Kearney, Kearney, NE 68849, USA; 5 Department of Biology, University of San Diego, San Diego, CA 92110, USA

**Keywords:** Buffalograss, Transcriptome, Next-generation sequencing

## Abstract

**Background:**

Buffalograss [*Buchloë dactyloides* (Nutt.) Engel. syn. *Bouteloua dactyloides* (Nutt.) Columbus] is a United States native turfgrass species that requires less irrigation, fungicides and pesticides compared to more commonly used turfgrass species. In areas where water is limited, interest in this grass species for lawns is increasing. While several buffalograss cultivars have been developed through buffalograss breeding, the timeframe for new cultivar development is long and is limited by a lack of useful genetic resources. Two high throughput next-generation sequencing techniques were used to increase the genomic resources available for buffalograss.

**Results:**

Total RNA was extracted and purified from leaf samples of two buffalograss cultivars. ‘378’ and ‘Prestige’ cDNA libraries were subjected to high throughput sequencing on the Illumina GA and Roche 454 Titanium FLX sequencing platforms. The 454 platform (3 samples) produced 1,300,885 reads and the Illumina platform (12 samples) generated approximately 332 million reads. The multiple k-mer technique for de novo assembly using Velvet and Oases was applied. A total of 121,288 contigs were assembled that were similar to previously reported Ensembl commelinid sequences. Original Illumina reads were also mapped to the high quality assembly to estimate expression levels of buffalograss transcripts. There were a total of 325 differentially expressed genes between the two buffalograss cultivars. A glycosyl transferase, serine threonine kinase, and nb-arc domain containing transcripts were among those differentially expressed between the two cultivars. These genes have been previously implicated in defense response pathways and may in part explain some of the performance differences between ‘Prestige’ and ‘378’.

**Conclusions:**

To date, this is the first high throughput sequencing experiment conducted on buffalograss. In total, 121,288 high quality transcripts were assembled, significantly expanding the limited genetic resources available for buffalograss genetic studies. Additionally, 325 differentially expressed sequences were identified which may contribute to performance or morphological differences between ‘Prestige’ and ‘378’ buffalograss cultivars.

## Background

Buffalograss [*Buchloë dactyloides* (Nutt.) Engel. syn. *Bouteloua dactyloides* (Nutt.) Columbus] is a turfgrass species native to the Great Plains region of the United States with exceptional drought, cold and heat tolerance. Buffalograss is often considered an ideal low input turfgrass species because it requires relatively less irrigation, fertility, and pesticide inputs to maintain an acceptable level of turfgrass quality compared to more commonly used turfgrass species [1]. With the increased frequency and duration of drought over the past few growing seasons, buffalograss demand by consumers is on the rise.

Buffalograss has a base haploid chromosome number of 10 and exists as a ploidy series ranging from diploid (2n = 20) to hexaploid (2n = 60). Diploids and tetraploids appear to be more southerly adapted, while hexaploids are found throughout the northern range of the Great Plains [2]. Buffalograss is a perennial species that is highly stoloniferous, forms a dense sod, has fine leaf texture, and is greyish green in color [1]. Buffalograss is also dioecious and thus is an obligate outcrossing, highly heterogeneous species which complicates cultivar development and genomic studies. Some challenges associated with buffalograss management are its intolerance of shade [3], short growing season in cooler climates [3], and susceptibility to certain pests [4,5]. Each of these traits is being addressed through breeding efforts to reduce the impact of these stresses on future buffalograss cultivars. Traditional buffalograss breeding strategies rely on genetic diversity among germplasm and the introgression of positive traits from one cultivar into another with improved turfgrass performance. The development of new buffalograss cultivars is a lengthy process that could be accelerated through the use of expanded genomic resources and molecular assisted breeding strategies.

Relative to the major agronomic food crops, there are few genomic resources available for studying buffalograss; for example, there are no buffalograss EST sequences published in GenBank [6] (accessed on 8/28/2013). To date, most of the genetic studies in buffalograss have been directed towards genetic marker development, resolving the taxonomy of buffalograss, and assessing genetic diversity of individuals among germplasm collections. For example, RAPD and isozyme markers were used to evaluate genetic relationships among two diploid buffalograss populations originating from central Mexico and two originating from Texas [7]. Sequence-related amplified polymorphic markers (SCAR) were used to assess genetic diversity among naturally occurring stands of buffalograss [8]. Both of these genetic marker studies observed a significant amount of genetic diversity among accessions collected from different geographic regions. The *matK*, *rbcL*, and *cob* genes were sequenced from 20 buffalograss accessions along with zoysiagrass (*Zoysia japonica* Steud.), bermudagrass [*Cynodon dactylon* (L.) Pers.], and blue grama [*Bouteloua gracilis* (H.B.K.) Lag. Ex Steud.] accessions [9]. The mitochondrial *cob* gene showed close association of the buffalograss cultivars ‘Bowie’ and ‘Density’ to the blue grama entry, while the plastid genes *matK* and *rbcL* clearly showed the buffalograss accessions were distinct from the other species studied.

Transcriptome sequence data of non-model organisms, such as buffalograss, is increasingly more accessible through the use of next generation sequencing strategies. Transcriptome sequencing is an ideal way of identifying trait specific genes, efficiently developing genetic markers, characterizing gene expression, and resolving gene networks, and is routinely applied to the study of organisms with little prior genomic information [10]. Next generation sequencing technologies are only just beginning to be applied to the study of turfgrass systems and have thus far focused primarily on improving our understanding of how turfgrasses responds to biotic and abiotic stress. For example, RNA-seq strategies were used to study the interaction between *Sclerotinia homoeocarpa*, the pathogen causing dollar spot disease, and creeping bentgrass (*Agrostis stolonifera* L.) whereby several genes were identified from either the host or the pathogen that were differentially expressed during infection [11]. Similarly, the *S. homoeocarpa* and creeping bentgrass disease interaction was studied using RNA-seq and transcriptome changes were identified [12]. A better understanding of genes involved in the pathogen-host interactions would facilitate the development of host resistance in future cultivars and help direct cultural practices to reduce the impact of disease. The SOLiD-SAGE technology was used to identify transcriptional changes in a red fescue (*Festuca rubra*) host infected with the *Epichloë festucae* endophyte [13]. Endophyte infection is often associated with improved stress tolerance of the host, and this study observed changes in host gene expression resulting from the presence of the endophyte. To the best of our knowledge, to date there have been no high-throughput sequencing experiments done on buffalograss; contributing to the limited genetic information available for studying this species.

In the present study, the transcriptome was sequenced of two buffalograss cultivars, ‘Prestige’ and ‘378’, known to differ in chinch bug resistance, ploidy level, and other turfgrass performance traits. The cDNA libraries were sequenced with both the Illumina GA and 454 Titanium FLX sequencing platforms, expanding buffalograss genetic resources. This is a valuable resource that turfgrass breeders and others in the turfgrass research community can use as a reference for comparative transcriptome studies, as a platform for genetic marker development, to characterize buffalograss variety differences, and to implement marker assisted breeding strategies for future cultivar development.

## Results

In total, 1,300,885 sequencing reads were generated on the 454 Titanium FLX sequencer, with 906,812 derived from ‘Prestige’ and the remaining 394,073 from ‘378’. The 454 sequencing reads had an average read length of 281 bp with a maximum read length of 669 bp. More than 159.3 M and 172.8 M Illumina GA 55 bp sequencing reads were generated for ‘Prestige’ and ‘378’, respectively. An average of 27.7 M reads was sequenced on the Illumina platform per sample. After strict quality filtering, 73.1 M and 67.6 M reads from ‘Prestige’ and ‘378’, respectively were used for sequence assembly. For ‘Prestige’, the combined Velvet/Oases k-mer assemblies with redundant sequences removed generated 265,590 transcripts with an average length of 899 bp and a maximum length of 18,330 bp. For ‘378’, the combined Velvet/Oases k-mer assemblies generated 241,129 transcripts with an average length of 835 bp and a maximum length of 11,681 bp (Table 1).

**Table 1:** Sequence statistics for transcripts with multiple k-mer assemblies combined and with BLAST matches to Ensembl commelinid reference proteins

**Assembly**	**No. of**	**Median**	**N50**	**Mean**	**Longest transcript**
	**transcripts**	**(bp)**	**(bp)**	**(bp)**	**(bp)**
Prestige multiple assemblies combined with Oases	265590	678	1353	899	18330
378 multiple assemblies combined with Oases	241,129	625	1253	835	11681
Prestige transcripts with BLAST match to Ensembl commelinid proteins	64,040	972	1499	1145	12,236
378 transcripts with BLAST match to Ensembl commelinid proteins	57,248	919	1422	1090	11,681

**Figure 1:**
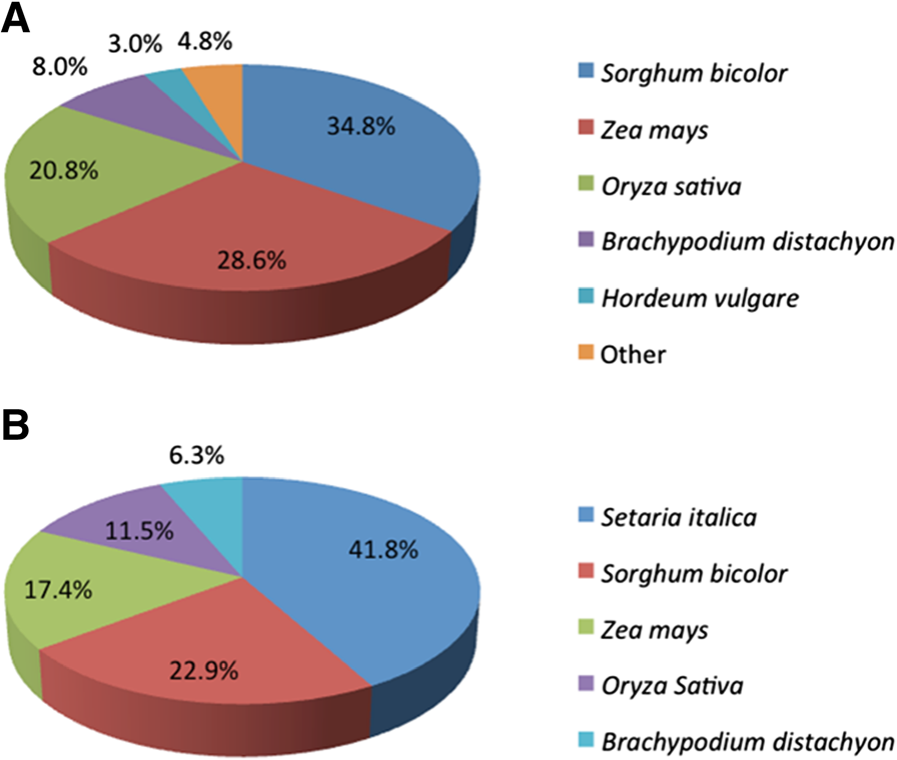
**Species distribution of the top BLAST hits for the ‘Prestige’ sequences.** Transcripts were compared to the NCBI nr database **(A)**, and also to model *Liliopsida* plants from Ensembl **(B)**.

Of the 265,590 ‘Prestige’ transcripts, 64,040 had significant BLASTx hits (e-value < 1E-10) to Ensembl commelinid plant protein sequences. Similarly, of the 241,129 ‘378’ transcripts, 57,248 had at least one BLASTx hit. The majority of transcripts had a significant level of sequence identity to foxtail millet (*Setaria italica*) proteins. The second closest reference species was *Sorghum bicolor*. A total of 17,512 unigene clusters were created within ‘Prestige’, and 16,743 clusters within the ‘378’ assembly. The NCBI non-redundant (nr) database lacks foxtail millet proteins, there are only 515 deposited. *Sorghum bicolor* is the most closely related species in the nr database based on BLASTp searches (Figure 1).

**Figure 2:**
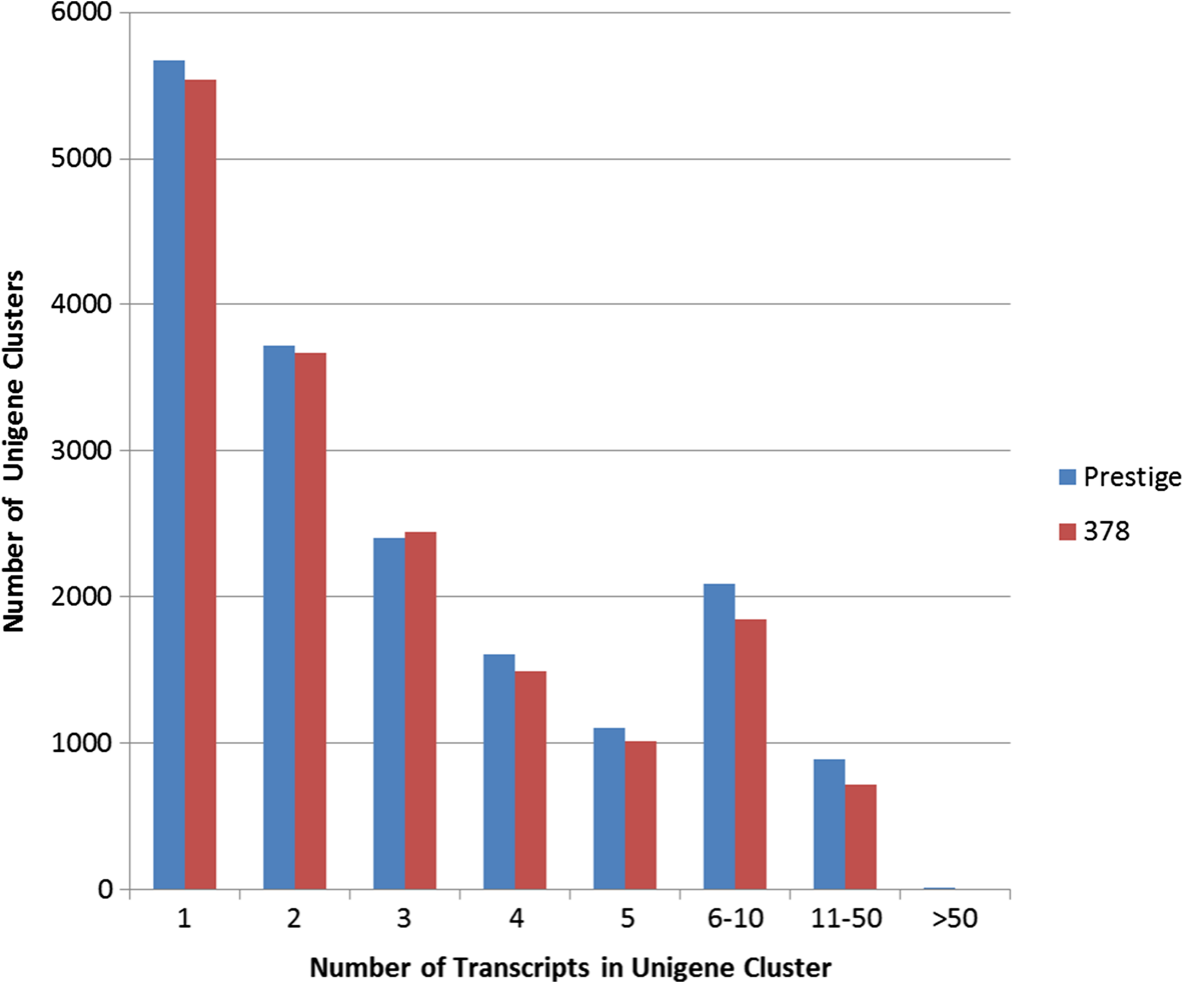
**Histogram of Unigene clusters in ‘Prestige’ and ‘378’ cultivars.** X-axis lists how many transcripts are in a unigene, and Y-axis lists how many unigenes are of that size.

While an average of 5,603 unigenes between the two transcriptomes only contained one transcript, many unigene clusters contained more than one sequence (Figure 2). These multiple transcript unigene clusters can represent transcription variants, allelic variants, closely related paralogues, misassembled transcripts, or transcripts that were fragmented due to low coverage. The latter case would require scaffolding to resolve based on alignments to reference transcripts, but was not conducted in these assemblies. The unigene containing the most transcripts, 170 transcripts in the ‘378’ assembly, was similar to the Si027417m.g gene in foxtail millet. This foxtail millet gene also represented the largest unigene cluster in ‘Prestige’. NCBI BLAST results indicate that this foxtail millet gene contains an nb-arc domain, which is common in plant disease resistance genes [14].

**Figure 3:**
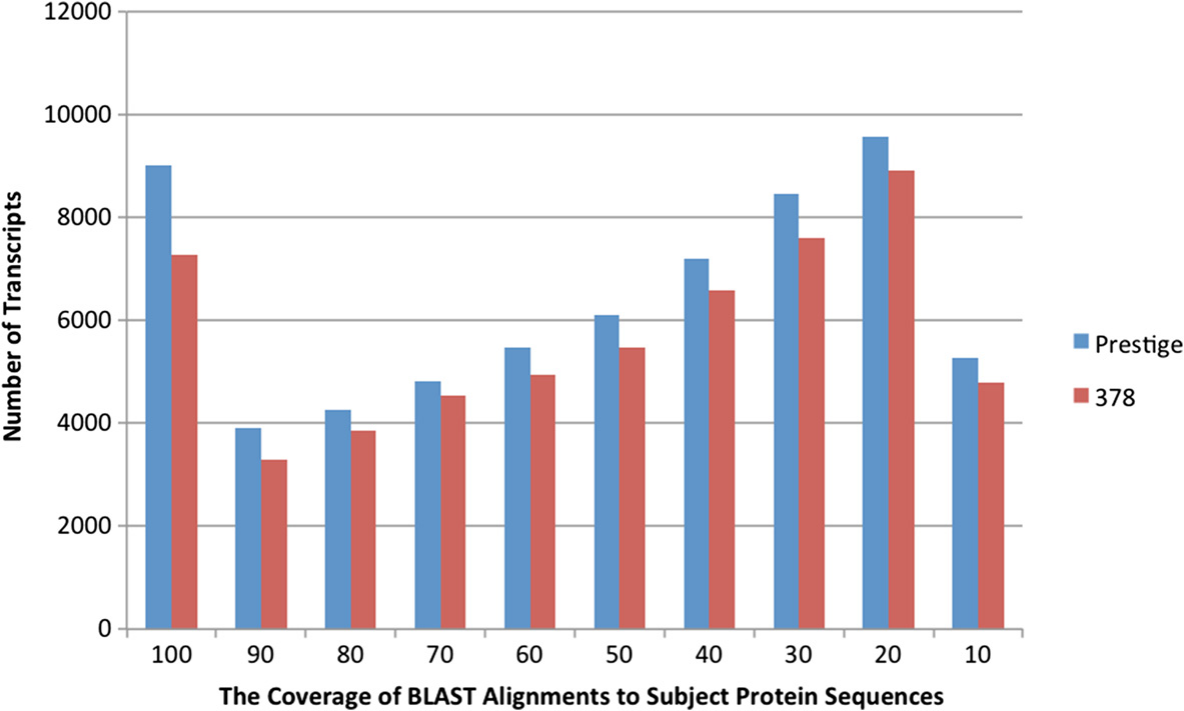
The number of transcripts versus the coverage of blast alignments to subject protein sequences.

‘Prestige’ transcripts shared sequence identity with 15,553 foxtail millet genes, which is 43.8% of the foxtail millet coding genes [15]. There is a high degree of synteny among the grasses and thus this percentage may be an initial indicator of how much of the buffalograss transcriptome was sequenced. The translated protein sequence of 3,658 transcripts in ‘Prestige’ and 2,988 transcripts in ‘378’ aligned to 100% of a reference protein via BLASTx, indicating that these were complete coding transcripts (Figure 3).

The predicted buffalograss proteins were searched against the NCBI nr database using BLASTp. The resulting BLASTp report was input to the BLAST2GO software. Gene ontology terms were assigned to 58,524 transcripts in ‘Prestige’ and 52,472 transcripts in ‘378’. Of these annotated sequences, 17,560 and 15,982 transcripts were assigned Enzyme Codes in ‘Prestige’ and ‘378’, respectively.

Original Illumina reads, per sample, were aligned to genotype specific assemblies. An average of 19.4 M reads from each sequenced sample successfully aligned to its corresponding transcriptome. Within each sample, an average of 5.5 M of these reads were unique alignments, while an average of 13.9 M mapped reads also aligned to other transcripts.

A reciprocal BLASTp search was performed to identify transcripts shared among the ‘Prestige’ and ‘378’ translated transcriptome libraries. A total of 19,861 reciprocal hits were identified. Of these reciprocal hits, 6,942 sequences had alignments where 100% of the ‘Prestige’ transcript length aligned to the ‘378’ transcript, or vice versa; these transcripts share the same protein length and sequencing reads covered the entire sequence length.

Using read counts from the previously mentioned Illumina read mapping, expression levels were generated for the transcripts having a reciprocal hit between cultivars. As mentioned in the Methods section, the focus of the gene expression analysis in this study was on transcripts where the majority of reads were uniquely aligned. Using the DESeq Bioconductor package, read counts were normalized using the estimateSizeFactors function, and the expression levels of the selected reciprocal hits were analyzed for statistical significance, p-value < 0.05 adjusted for multiple testing. There were 325 differentially expressed genes between the two cultivars. Of these genes, 171 had higher expression in ‘Prestige’, and 154 genes had higher expression in ‘378’.

**Figure 4:**
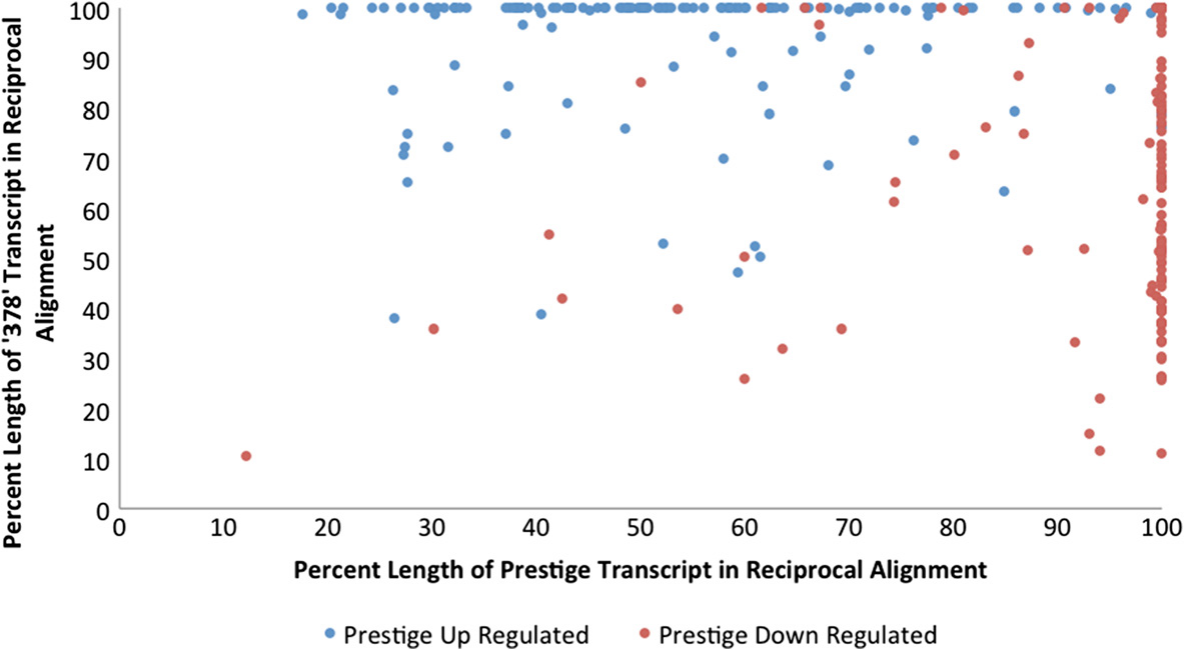
**Scatter plot displaying percent length of ‘Prestige’ transcripts aligning to percent length of ‘378’ transcripts.** Transcripts shown are those that were a reciprocal match and significant expression change was detected.

Expressed genes in which at least 75% of the length of the ‘Prestige’ transcript aligned to at least 75% of the reciprocal matched ‘378’ transcript were further analyzed (Figure 4; Table 2). During the process of finding reciprocal sequences among the two genotypes, it was observed that several transcripts had no significant BLAST match to any transcript in the other genotype. This suggests that the gene is not expressed in the other genotype in these samples, it wasn’t expressed enough to be assembled, or does not exist in the other genome. Read counts for these transcripts were analyzed, and any sequence where all of its matching reads were unique alignments was considered valid (Table 3).

**Table 2:** List of significantly expressed genes between Prestige and 378 cultivars

**Prestige**	**Transcript**	**Prestige read**	**378 read**	**Log2 fold**	**P adjusted value**	**Description**
**transcript**	**length (bp)**	**numbers**	**numbers**	**change**	**(multiple testing FDR)**	
preC_72587	1987	2990.6	211.6	−3.82	6.79E-20	transcription factor-like protein dpb-like
preC_221736	1868	1456.9	133.7	−3.45	1.32E-13	transferring glycosyl
preC_91046	1253	286.7	35.0	−3.04	5.40E-08	wd repeat-containing protein 76-like
preC_256602	374	29.4	3.8	−2.94	2.49E-02	protein kinase family protein
preC_230932	507	135.8	18.5	−2.88	7.85E-03	solute carrier family 25 member 44-like
preC_165762	589	60.9	9.4	−2.69	1.17E-03	transmembrane protein 97
preC_90290	713	43.1	6.8	−2.67	1.98E-02	peptidase c48 domain family protein
preC_211115	714	386.1	61.9	−2.64	3.50E-05	potassium transporter
preC_81161	1860	1697.7	301.4	−2.49	5.86E-03	1-acyl-sn-glycerol-3-phosphate acyltransferase 4
preC_261585	1410	455.2	92.2	−2.30	3.51E-02	ankyrin-like protein
preC_231933	216	64.6	275.3	2.09	1.51E-05	chalcone isomerase-like protein
preC_101008	1056	153.7	673.8	2.13	3.37E-03	ras-related protein raba3-like
preC_26965	294	100.4	448.7	2.16	4.51E-06	tata-binding protein2
preC_127066	1068	41.4	206.6	2.32	7.43E-03	dna cross-link repair protein snm1
preC_134037	488	15.8	120.8	2.93	4.87E-07	ent-kaurenoic acid partial
preC_41455	462	15.6	119.7	2.94	3.17E-06	dna helicase
preC_241217	352	19.7	154.3	2.97	2.86E-07	f-box kelch-repeat protein skip6-like
preC_217560	350	8.3	68.0	3.03	2.93E-02	sister of ramosa partial
preC_42285	748	57.3	1020.9	4.16	2.86E-04	cationic peroxidase 1-like
preC_226473	1365	173.6	4669.0	4.75	4.77E-25	pentatricopeptide repeat-containing protein

Listed are top 10 up-and down-regulated genes with the removal of hypothetical-or uncharacterized-proteins.

**Table 3:** Hypothetical cultivar-specific genes based upon the reciprocal blast

**Transcript**	**Transcript**	**Average read**	**Description**
	**length (bp)**	**numbers**	
Prestige			
preC_247169	647	206.0	dna repair and recombination protein
preC_214648	316	61.5	maize proteinase inhibitor
preC_236041	545	54.0	copper transporter 1
preC_262069	551	33.3	proteasome assembly chaperone 2
preC_256520	237	28.2	e-cadherin binding protein
preC_167144	650	23.7	cle family 306 protein
preC_256357	190	21.2	zinc finger family expressed
preC_128843	234	15.2	pentatricopeptide repeat-containing protein
preC_231580	353	14.8	s-receptor kinase
preC_169805	381	13.5	protein epidermal patterning factor 2-like
378			
378C_71451	258	278.7	snare-like protein
378C_151851	237	108.5	tubulin-specific chaperone d-like
378C_238144	303	84.2	subtilisin-like protease
378C_152448	174	70.7	lin1 protein
378C_199252	175	51.8	craniofacial development protein 1-like
378C_223520	325	47.0	pollen-specific protein like
378C_24916	192	18.5	trab domain-containing
378C_235655	173	16.8	nicotinate-nucleotide pyrophosphorylase
378C_202598	275	15.3	growth-regulating factor 2
378C_135613	214	10.2	serine threonine-protein kinase ctr1-like

Listed are the top 10 most highly expressed from each cultivar with the removal of sequences of vague descriptions. Average expression is a measure of reads mapped per sample.

**Figure 5:**
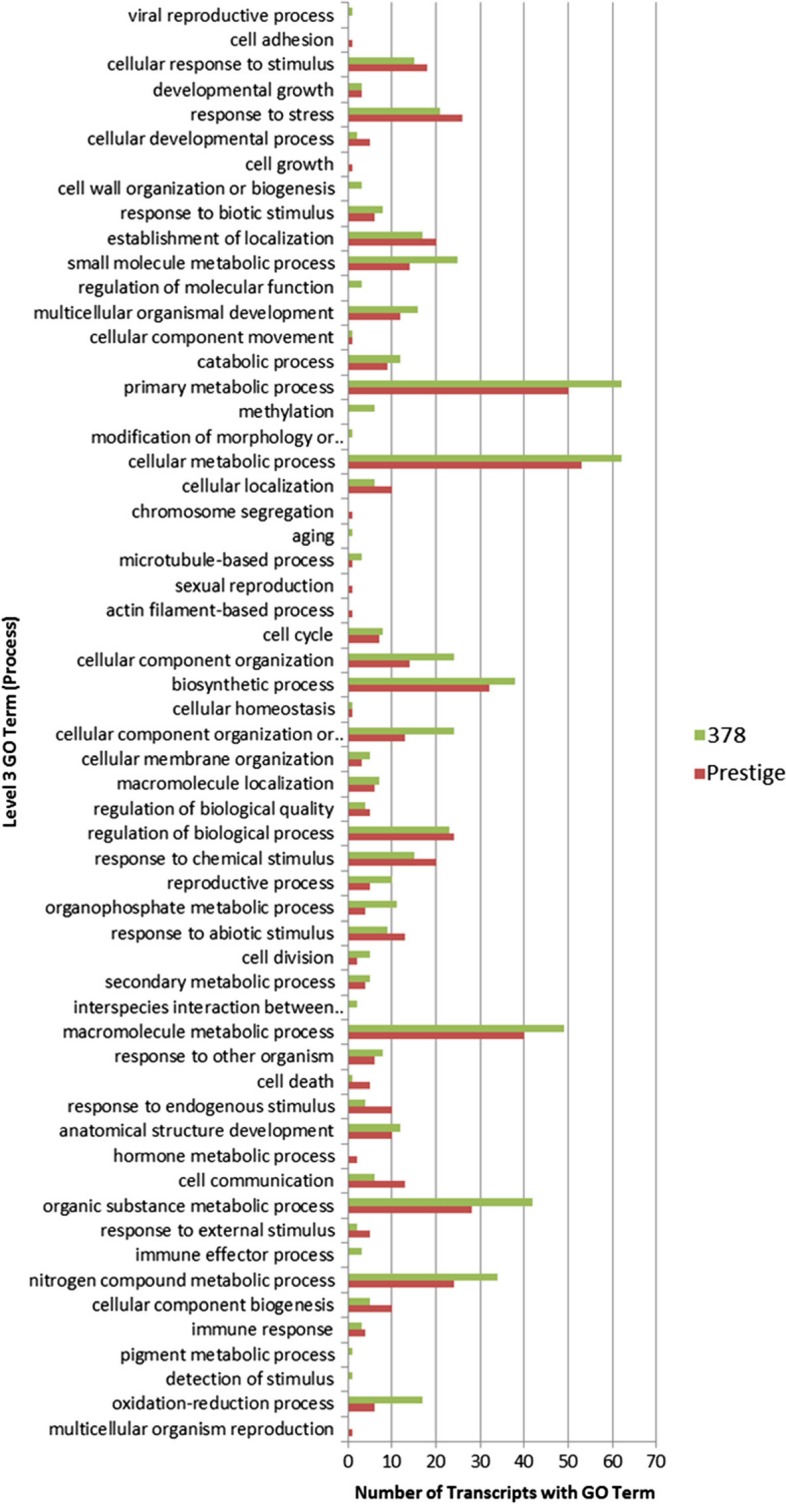
**Number of significantly expressed genes in each of the Level 3 GO biological processes.** Transcripts with higher expression in ‘Prestige’ than in ‘378’ are in red. Transcripts with higher expression in ‘378’ than in ‘Prestige’ are in green.

GO terms were assigned to all 325 differentially expressed genes between the two cultivars, including those having incomplete alignments to a reciprocal sequence. Quantification of Level 3 gene ontology terms was collected for these transcripts (Figure 5).

Gene ontology terms were used to select sequences related to stress and immune response. The parental gene ontology terms for “response to stress” (GO:0006950), “response to other organism” (GO:0051707), and “immune response” (GO:0006955) were found in 27 up-regulated ‘Prestige’ genes, and in 24 down-regulated ‘Prestige’ genes. A number of these genes have previous research linking them to defense and immune responses (Table 4).

**Table 4:** Significantly expressed genes that have previous research evidence of stress response

**Prestige transcript**	**Transcript length (bp)**	**Prestige read numbers**	**378 read numbers**	**Log2 fold change**	**p-value**	**Description**	**References**
preC_246578	2200	177.09	7.79	−4.51	3.10E-02	nb-arc domain-containing protein	[16-18]
preC_139482	693	31.78	2.73	−3.54	1.27E-03	serine threonine kinase (U-box domain)	[19-22]
preC_221736	1868	1456.86	133.67	−3.45	1.32E-13	transferring glycosyl	[23-25]
preC_224469	1554	162.26	16.25	−3.32	8.85E-03	nbs-lrr class disease resistance protein	[16-18]
preC_86410	2051	362.42	60.17	−2.59	1.15E-05	dna repair protein xrcc2-like protein	[26,27]
preC_231454	1257	523.56	1972.11	1.91	2.99E-05	uracil phosphoribosyltransferase	[28]
preC_143034	881	111.18	449.67	2.02	2.32E-05	chloroplast processing peptidase	[29]
preC_249539	576	30.03	132.51	2.14	2.26E-04	gamma-glutamyl transpeptidase 1	[30-32]
preC_127066	1068	41.38	206.6	2.32	7.43E-03	dna cross-link repair protein snm1	[33]
preC_42285	748	57.27	1020.91	4.16	2.86E-04	cationic peroxidase 1-like	[34,35]

Genes containing GO terms “response to stress”, “immune response” and “response to other organism” were selected.

## Discussion

There are limited genetic resources available for studying buffalograss, however with current next generation sequencing and de novo assembly strategies, high throughput sequencing can help bridge this buffalograss knowledge gap. In the present study, 121,288 high quality transcripts were reconstituted from ‘Prestige’ and ‘378’ buffalograss cultivars, utilizing a combination of Illumina GA and Roche 454 Titanium FLX sequencing. Transcripts were found to be differentially expressed between samples of the same genotype collected at different times. Physiological differences are expected to occur between the two sampling times for a given genotype. Since the scope of this research was to expand buffalograss genetic resources and characterize differences between ‘378’ and ‘Prestige’, differences occurring within a genotype between the two time points were not examined.

When compared to the NCBI nr database, approximately 50% of the transcripts had BLASTp hits to *Sorghum bicolor* proteins, while only 5% of the transcripts had hits to Brachypodium (*Brachypodium distachyon*). Brachypodium is often considered a model for the study of grasses, but in this instance was the least informative when compared to the buffalograss transcripts (Figure 1). There are approximately three times as many *S. bicolor* sequences in the nr database compared to Brachypodium which may help explain why more *S. bicolor* hits were returned. Additionally, both *S. bicolor* and buffalograss are warm season, C4, grasses while Brachypodium is a cool season, C3, grass and therefore it is not surprising that buffalograss sequences were more similar to *S. bicolor*.

The two buffalograss genotypes used in this study, ‘Prestige’ and ‘378’, are known to differ in terms of their resistance to chinch bugs, an important insect pest on buffalograss [36][37]. Previous data suggests that oxidative enzymes play a role in chinch bug resistance in buffalograss [38]. For example, the chinch bug resistant cultivar ‘Prestige’ had higher peroxidase activity under both uninfested and chinch bug-infested conditions compared to the susceptible ‘378’ [36]. In the present study, 325 differentially expressed genes between these two genotypes were identified (Table 2). If ‘Prestige’ is predisposed for chinch bug resistance, genes expressed higher in ‘Prestige’ compared to susceptible genotypes may be involved in the resistance mechanism. Similarly, genes more highly expressed in the susceptible cultivar may confer susceptibility.

The differences in gene expression between genotypes may account for some of the performance differences among these cultivars. Selecting expressed genes based on gene ontology terms for stress and immune response highlights a potential starting point for understanding these mechanisms. Several of these genes have been researched in regards to stress tolerance. For example, the nb-arc domain-containing and the nbs-lrr class of proteins are known to be involved in a plant’s defense response. The nucleotide binding and amino-terminal domains contain a nucleotide-binding site and may act as a molecular switch, regulating specific downstream pathways. Large unigene clusters from both ‘378’ and ‘Prestige’ were similar to the nb-arc gene, Si027417m.g, from foxtail millet. The preC_246578 transcript has higher average read counts in ‘Prestige’ (177.09 average reads) compared to ‘378’ (7.79 average reads), representing a−4.51 log2 fold difference in expression (p-value = 3.1E-02) between the two cultivars (Table 4). The transcript, preC_224469, also had higher expression in ‘Prestige’ than ‘378’ (log2 fold change = −3.32; p-value = 8.85e-03). The preC_224469 transcript is predicted to be a member of the nbs-lrr family of genes. The leucine-rich repeat (lrr) domain may act as the signaling molecule and be involved in recognizing early signs of a pathogen attack [16]. The predicted coding sequences from these transcripts contain nb-arc domains. The nb-arc containing proteins are most often associated with disease resistance. There has been limited research characterizing differences in disease resistance of ‘Prestige’ and ‘378’, so it would be interesting to test if ‘Prestige’ is more resistant to disease relative to ‘378’ and monitor expression of these genes during host-pathogen interactions.

Receptor like kinases also contain an lrr domain and may be involved in early pathogen attack recognition and regulate the level of response to pathogen attack, playing a role in triggering early defense response signaling mechanisms [19]. The transcript, preC_231580, is a serine threonine kinase and had higher expression in ‘Prestige’ relative to ‘378’ (log2 fold change = −3.54, p-value = 1.27e-03).

Glycosyl transferases are also involved in stress-induced plant response and show elevated expression in response to several signaling molecules including hydrogen peroxide [23]. For example, expression of two glycosyltransferases, UGT73B and UGT73B5, were important for Arabidopsis resistance to *Pseudomonas*[23]. The relative higher expression of a transferring glycosyl, preC_221736 (Table 4), in ‘Prestige’ compared to ‘378’ (log2 fold change = −3.45, p-value = 1.32e-13) is of particular interest since response to oxidative stress may be one mechanism conferring resistance to chinch bugs in ‘Prestige’ [38].

The majority of the defense response genes identified in this study are not directly linked to insect resistance, however this study characterizes differences between ‘Prestige’ and ‘378’ which may facilitate a better understanding of host pest interactions in future studies. The majority of the previously mentioned defense response genes are associated with disease resistance. Buffalograss is most commonly grown throughout the Great Plains region of the United States [1] and since buffalograss grows in this relatively arid region of the country, there is less disease pressure than in more humid regions. As a result, limited research has been done to evaluate disease resistance of ‘Prestige’ and ‘378’.

Since two distinct buffalograss genotypes were sequenced here, genotypic differences such as single nucleotide polymorphisms, copy numbers of simple sequence repeats, insertion/deletions, and transposable element insertion polymorphisms [39] could be exploited to develop genetic markers for cultivar discrimination or associated with a trait of interest that differs between the two genotypes. In addition, since the sequences presented here are based on expressed transcripts, any genetic markers developed from these sequences are, by nature of the study, gene-based and ultimately more valuable for future molecular-based cultivar development strategies. Since for example, ‘378’ and ‘Prestige’ are known to differ in chinch bug resistance, the identification of polymorphic homologous sequences in these plants is a first step at developing markers to use in a marker assisted breeding scheme to improve chinch bug resistance. This research would need further investigation to characterize the markers in a broader germplasm base that has been evaluated for chinch bug resistance.

## Conclusions

This is the first report of transcriptome sequencing of Buffalograss [*Buchloë dactyloides* (Nutt.) Engel. syn. *Bouteloua dactyloides* (Nutt.) Columbus], the most widely used native turfgrass species in the United States. Transcriptomes of buffalograss cultivars ‘378’ and ‘Prestige’ were sequenced by Illumina GA and Roche 454 Titanium FLX sequencing platforms and 121,288 high quality transcripts were assembled. There were 15,553 ‘Prestige’ transcripts that had significant BLAST hits to foxtail millet (*Setaria italica*) which could be useful for future comparative genetic studies between these species. Transcriptional profiling revealed 325 differentially expressed genes between ‘378’ and ‘Prestige’ and may in part help explain cultivar differences. At the time of this study, there were no reported buffalograss EST sequences in NCBI and only 34 nucleotide sequences (accessed August 28th, 2013), so this study significantly expands on the limited genetic resources available for studying buffalograss. The data presented here will act as a platform for genetic marker development, a basis for marker assisted breeding strategies, and a reference for future transcript expression studies.

## Methods

### Sample preparation and sequencing

Vegetative plugs (10.6 cm diameter × 8 cm deep) of ‘378’ and ‘Prestige’ were collected from the University of Nebraska Agricultural Research and Development Center, near Mead, NE. Individual stolons from a single plant of each cultivar were planted in SC-10 Super Cell single cell 3.8 cm diameter × 21 cm deep cone-tainers (Stuewe & Sons, Inc. Corvallis, OR). The clonal ramets were used for the sequencing studies. The soil mixture was a ratio of 2:1:3:3 sand, soil, peat, and perlite. Buffalograss plants were watered and fertilized (20 N-10P-20 K soluble) as needed. Plants were maintained at a temperature of 24 ± 3°C and a 16 h photoperiod under 400-watt high-intensity discharge lamps.

The experiment was designed as a 2 × 2 factorial with two buffalograss genotypes (‘Prestige’ and ‘378’) and two distinct time points seven days apart. The study was arranged as a randomized complete block design with six replications. Buffalograss leaf samples were collected from three replicates of ‘Prestige’ and three replicates of ‘378’. A similar set of leaf samples were collected seven days later. For each cultivar, two separate time points were used to minimize transcriptional variation introduced by changes in the environment, growth stage, or physiological differences of the plants. Total RNA was isolated from the leaf samples and all 12 were prepared for sequencing on the Illumina GA sequencing platform. A single leaf sample of ‘Prestige’ and a single leaf sample of ‘378’ collected on the first sampling date, along with a single sample of ‘Prestige’ collected on the second sampling date were prepared for sequencing on the Roche 454 Titanium FLX sequencer. Leaf tissue was collected for RNA extraction and immediately frozen in liquid nitrogen and stored at −80°C. Four 100 mg leaf tissue samples for each of the 12 buffalograss samples were used as starting material in the RNA extraction procedure and later merged such that there was one composite RNA sample per buffalograss sample. In short, mRNA was then extracted using the FastTrackMAG maxi kit (Invitrogen #K158002) and cDNA was created using the QuantiTect Whole Transcriptome kit (Qiagen #207043). The cDNA was cleaned up using the QiAamp DNA Blood mini kit (Qiagen #51104) before submitting the samples for sequencing.

The leaf tissue mRNA samples were sequenced on the 454 Titanium FLX platform and each sample used one half picotiter plate. A total of twelve samples were sequenced on an Illumina GA Sequencer; these samples consisted of 3 replications of each genotype collected at the first and second time points. One Illumina flowcell lane was used for each sample.

### Data filtering and de novo assembly

A strict quality filtering pipeline was used to select reads for assembly. 454 reads were quality filtered and polyA tail trimmed using Newbler 2.6 software with the “-cdna–tr” options [40]. Redundant reads sharing 100% identity were removed using CD-HIT-454 [41]. Reads longer than 75 bp were selected as “long” reads for the Velvet/Oases assembly process. These reads were used as reference sequences in the Velvet assembler.

Illumina reads containing at least one base with a quality score below 10 were removed, as well as duplicate reads using FastQ program. PolyA tail trimming was performed by removing reads with at least half of the read length containing all adenines or thymines. Reads containing adapter sequences were identified and removed with Tagdust [42].

Separate transcriptomes were assembled for each genotype. Due to the polyploid nature of these plants and a potential high level of intra-organism and inter-organism variation, such as genome rearrangements or paralogue genes unique to one genotype, we decided to not combine genotype reads. Combining reads from both genotypes could potentially complicate the assembly process and create inaccurate transcripts. Assembly was performed using Velvet/Oases software [43]. Multiple assemblies were created per genotype, using odd k-mer values 27-51. Previous studies have shown that using multiple assemblies, at varying k-mer values, captures more lowly expressed transcripts when compared with a single k-mer assembly [44]. Combined transcripts from the multiple k-mer assemblies were run through the CD-HIT-EST program to remove redundant transcripts sharing 100% identity [41].

### Functional annotation

Transcripts from the two genotype specific assemblies were aligned to a database containing all Ensembl proteins from *Brachypodium distachyon*, *Oryza sativa*, *Setaria italica*, *Sorghum bicolor*, and *Zea mays* using BLASTx, e-value threshold of 10e-10. Initial BLASTx results showed that *Setaria italica* produced the majority of best BLASTx hits, so this reference species was chosen as a beginning reference for annotation. BLASTx was again used to compare assembled transcripts to only *Setaria italica* proteins. Transcripts were assigned to unigene clusters based on their best gene hit within a reference species. Any transcripts without a BLASTx hit to *Setaria italica* were compared to a database of *Brachypodium distachyon*, *Oryza sativa*, *Sorghum bicolor*, and *Zea mays* proteins. These transcripts were also assigned to unigene clusters based on their best BLASTx hit to this secondary reference protein database. The Ensembl gene accession names were used to label the unigene clusters. Any remaining transcripts not having a significant BLASTx hit to Ensembl plant proteins were removed from the transcriptome and not used in downstream analysis.

Using the BLASTx reports from the previous Ensembl protein search, translated open reading frames were extracted from the transcripts using the OrfPredictor software [45]. These extracted protein sequences were input to CD-HIT with 100% identity threshold to remove transcripts with identical protein translations. The remaining buffalograss protein sequences were compared to the NCBI nr database using BLASTp (e-value threshold of 10E-10). The BLASTp results were input into the BLAST2GO program to assign sequence descriptions, gene ontology terms, and enzyme commission numbers [46].

Extracted ORF sequences from the ‘Prestige’ assembly were compared to ‘378’ sequences via BLASTp (e-value 10E-10), and vice versa. If two transcripts from both genotypes had a reciprocal best BLASTp hit to each other, these two transcripts were assigned the same reciprocal hit ID number and considered to be the same gene in the two genotypes. If a reciprocal hit transcript of one genotype only aligns to a portion of the reciprocal hit in the other genotype (<75% of the length of either transcript) it was discarded. These shorter alignments can occur because of sequence variation, low expression, and incomplete assembly. The 75% cutoff was chosen to limit the occurrence of false positives and to return longer reciprocal hits which could be useful in future comparative genetic studies between these cultivars.

### Expression analysis

All genotype specific Illumina reads, including reads not used in the assembly process, were aligned to the genotype specific transcriptome using Bowtie alignment software [47]. Read counts for transcripts with a reciprocal match to the other transcriptome were counted and extracted for gene expression analysis, per replicate and time point of sample. Due to the polyploidy of the genomes, and a high number of closely related paralogues within plants, a portion of aligned reads will align to more than one transcript. These “multi-mapped” reads can lead to false read counts for many transcripts. For gene expression analysis, we only examined transcripts with a reciprocal hit in the other genotype where >75% of the aligned reads were unique alignments, not aligning to any other transcripts. Per replicate and time point, the sum of uniquely aligned reads was output to a matrix. The two time points were used to minimize transcriptional variation for each genotype introduced by environmental changes at the time the samples were taken and physiological differences. Therefore all six samples within a genotype were treated as replicates for the statistical analysis. Relative expression based on read counts was used instead of RPKM values because the transcriptomes varied by sequence number and sequence lengths. The matrix of read counts was input into DESeq R Statistical package to identify transcripts with significant expression between genotypes (FDR < 0.05) [48]. The read counts of transcripts having no significant BLAST hit to the other genotype were also examined, as these may represent transcripts not assembled or expressed in the other transcriptome. Differentially expressed sequences were examined via BLAST and the NCBI nr database to determine if they are plant proteins or results of metatranscriptome contamination (e.g. bacteria or fungi).

## Availability of supporting data

The data sets supporting the results of this article are available in the BioProject (BioProject:PRJNA207980) repository of the National Center for Biotechnology Information, http://www.ncbi.nlm.nih.gov/bioproject/?term=PRJNA207980.

## Competing interests

The authors declare that they have no competing interests.

## Authors’ contributions

The project was conceived by THM and PT. MW conducted de novo transcriptome assembly and transcriptional profiling. MW, KLA and GL oversaw bioinformatics analyses. LB, THM, PT, and KLA interpreted biological relevance of results. MW and KLA wrote the manuscript and all authors read, edited, and approved the manuscript.
